# Editorial: Fertility Preservation in Asia

**DOI:** 10.3389/fendo.2020.603213

**Published:** 2021-01-08

**Authors:** Jung Ryeol Lee, Seido Takae, Nao Suzuki

**Affiliations:** ^1^ Department of Obstetrics and Gynecology, Seoul National University Bundang Hospital, Seongnam, South Korea; ^2^ Department of Obstetrics and Gynecology, Seoul National University College of Medicine, Seoul, South Korea; ^3^ Department of Obstetrics and Gynecology, St. Marianna University School of Medicine, Kawasaki, Japan

**Keywords:** fertility preservation, Asia, oncofertility, cancer, Asian countries

In recent years, fertility preservation (FP) has become an important concern for cancer patients, and also for women seeking protection against future infertility due to aging or other causes. The Asian Society for Fertility Preservation (ASFP) was founded in 2015 by experts from 14 countries (Australia, China, Hong Kong, India, Indonesia, Japan, Korea, Pakistan, Philippines, Singapore, Taiwan, Thailand, Turkey, and Vietnam) to promote FP science and practice. In Asia, awareness of the importance of FP has gradually increased with new developments in clinical practice and research. The purpose of this editorial is to introduce a Research Topic consisting of articles published in a Research Topic of the journal *Frontiers in Endocrinology*, entitled “Fertility Preservation in Asia”.

The ASFP is the first initiative by experts across Asia to promote FP research and clinical application. Its mission is to raise awareness of FP among healthcare professionals and the public, improve technical skills, and keep healthcare practitioners informed about the latest developments in the field and research environment (Harzif et al.). The ASFP strives to develop FP programs in Asian countries through exchanges between countries, conferences, and educational programs such as hands-on workshops. Future goals include development of a strategy to promote multidisciplinary approaches between practitioners, policy creation, and advancement of a referral system to benefit patients (Harzif et al.).

In the following section, we introduce two review papers addressing FP practices in Asian countries. Takae et al. investigated currently available FP options in Asian countries for child and adolescent (CAYA) cancer patients. In November 2018, a questionnaire survey of founding members of the ASFP was conducted to identify FP barriers for CAYA cancer patients, and the results indicated that most Asian countries could provide FP treatment. Among the 11 Asian countries responding to the survey, five had organizations or academic societies promoting FP, and Australia, Japan, and Korea had organizations specializing in FP. In contrast, China and Indonesia maintained committees or branches of large academic societies focused on reproductive or maternal-child health medicine. Furthermore, Hong Kong and the Philippines are planning to establish organizations or academic societies specializing in FP. However, the lack of experience and an established framework for FP promotion are factors that may hinder its implementation, and more constructive discussion is needed to support FP for CAYA cancer patients in Asian countries. Harzif et al. suggested that the particular situations existing in each country should be addressed to provide optimal FP development and fulfill the needs of patients and physicians. Necessary conditions for implementing FP in various Asian countries were described by the authors of the abovementioned studies.

Cryopreservation programs for oocytes or tissues from cancer patients are available in most countries that have joined the ASFP. In Korea, a live birth resulting from vitrified-warmed oocytes harvested from a chronic myeloid leukemia patient was reported (1). In Japan, reports showed that auto-transplantation of ovarian tissue after cryopreservation successfully resulted in live births (2). And in 2013, cases of pregnancies and live births were reported in Singapore after ovarian cancer patients underwent oophorectomy and IVM oocyte cryopreservation (Harzif et al.). Since FP programs have only recently been initiated, additional reports of pregnancies and live births in similar cases are likely to occur. We anticipate that the review articles discussed above will become useful resources to help overcome barriers and encourage future development of FP programs in Asian countries.

Recent developments in “omics” have accelerated research on genes and RNAs related to FP. Tu et al. revealed that microRNAs of granulosa cells (GCs) are essential regulators of GC function under physiological and pathological conditions. The authors suggested that specific microRNAs could be targeted in the future for treatment of ovarian-related diseases such as polycystic ovary syndrome (PCOS), premature ovarian failure (POF), and granulosa cell tumors (GCT). Choi et al. found that expression levels of various genes related to DNA double-strand break (DSB) repair decreased in women with endometriosis, and consequently FP should be considered in these cases since impaired DSB repair gene expression may reduce ovarian reserve.

This special issue introduces various FP-related diagnostic, treatment, and prognostic studies performed by Asian research groups. Son K-A et al. analyzed the association between BRCA mutations and anti-Mullerian hormone (AMH) levels in young breast cancer patients using linear and logistic regression analysis. As a result of the analysis, breast cancer patients with BRCA mutations showed significantly lower serum AMH levels, and the authors suggested that young breast cancer patients should consider preserving fertility more actively. Son W-Y et al., in a review paper on *in vitro* maturation (IVM) of human oocytes, suggested the possibility of successful IVM for FP in women at risk of losing ovarian function. The authors also explained the benefits of IVM associated with oocyte vitrification. Kim et al. reported that using autologous platelet-rich plasma (PRP) in patients with refractory thin endometrium improved implantation, pregnancy, and live birth rates, introducing a possible new treatment for patients with infertility due to endometrial factors.

Two articles on infertility are also covered in this issue. Che et al. reported that cardiovascular disease and recurrent miscarriage shared risk factors, and some cardiovascular disease-related candidate genes were associated with recurrent miscarriage. Cai et al. showed that higher thyroid-stimulating hormone (TSH) levels were related to hyperandrogenism in women with euthyroid PCOS independent of age, BMI, and thyroid autoimmunity. Although it is widely known that thyroid dysfunction can lead to infertility, this report highlights the relationship between TSH and a specific disease.

These articles collectively cover current developments and predict future directions for FP in Asia. Patient demand for FP is expected to increase in the future since it is a key area of reproductive medicine. We expect that the articles in this Research Topic will serve as an important cornerstone to mark the beginning of future FP development, and also to stimulate FP research and practice in Asia and worldwide.

## Author Contributions

JL: draft, revision, and final approval of the manuscript. ST: draft, revision, and final approval of the manuscript. NS: draft, revision, and final approval of the manuscript. All authors contributed to the article and approved the submitted version.

## Conflict of Interest

The authors declare that the research was conducted in the absence of any commercial or financial relationships that could be construed as a potential conflict of interest.
